# Genome of the R-body producing marine alphaproteobacterium *Labrenzia alexandrii* type strain (DFL-11^T^)

**DOI:** 10.4056/sigs.3456959

**Published:** 2013-02-25

**Authors:** Anne Fiebig, Silke Pradella, Jörn Petersen, Orsola Päuker, Victoria Michael, Heinrich Lünsdorf, Markus Göker, Hans-Peter Klenk, Irene Wagner-Döbler

**Affiliations:** 1 Leibniz Institute DSMZ – German Collection of Microorganisms and Cell Cultures, Braunschweig, Germany; 2 HZI – Helmholtz Center for Infection Research, Braunschweig, Germany

**Keywords:** aerobe, motile, symbiosis, dinoflagellates, photoheterotroph, high-quality draft, *Alexandrium lusitanicum*, *Alphaproteobacteria*

## Abstract

*Labrenzia alexandrii* Biebl *et al*. 2007 is a marine member of the family *Rhodobacteraceae* in the order *Rhodobacterales*, which has thus far only partially been characterized at the genome level. The bacterium is of interest because it lives in close association with the toxic dinoflagellate *Alexandrium lusitanicum*. Ultrastructural analysis reveals R-bodies within the bacterial cells, which are primarily known from obligate endosymbionts that trigger “killing traits” in ciliates (*Paramecium* spp.). Genomic traits of *L. alexandrii* DFL-11^T^ are in accordance with these findings, as they include the *reb* genes putatively involved in R-body synthesis. Analysis of the two extrachromosomal elements suggests a role in heavy-metal resistance and exopolysaccharide formation, respectively. The 5,461,856 bp long genome with its 5,071 protein-coding and 73 RNA genes consists of one chromosome and two plasmids, and has been sequenced in the context of the **M**arine **M**icrobial **I**nitiative.

## Introduction

Strain DFL-11^T^ (= DSM 17067 = NCIMB 14079) is the type strain of *Labrenzia alexandrii*, a marine member of the *Rhodobacteraceae* (*Rhodobacterales*, *Alphaproteobacteria*) [1]. Strain DFL-11^T^ was isolated from single cells of a culture of the toxic dinoflagellate *Alexandrium lusitanicum* maintained at the Biological Research Institute of Helgoland, Germany [1]. *L. alexandrii* is the type species of the genus *Labrenzia*, which currently also harbors a couple of species (*L. aggregata*, *L. alba* and *L. marina*) that were previously classified in the genus *Stappia* [1]. Biebl *et al*. 2007 [1] did not provide a formal assignment of the genus *Labrenzia* to a family, but their phylogenetic analysis placed *Labrenzia* with high support within a clade also comprising *Nesiotobacter, Pannonibacter*, *Pseudovibrio, Roseibium* and *Stappia*, genera which at that time were either not formally assigned to a family or to *Rhodobacteraceae* [2]. Other analyses [3] indicate that the entire clade should not be placed within *Rhodobacteraceae*, but an alternative taxonomic arrangement has, to the best of our knowledge, not yet been published. Here we present a summary classification and a set of features for *L. alexandrii* DFL-11^T^ including so far undiscovered aspects of its ultrastructure and physiology, together with the description of the high-quality permanent draft genome sequence and annotation.

This work is part of the Marine Microbial Initiative (MMI) which enabled the J. Craig Venter Institute (JCVI) to sequence the genomes of approximately 165 marine microbes with funding from the Gordon and Betty Moore Foundation. These microbes were contributed by collaborators worldwide, and represent an array of physiological diversity, including carbon fixation, photoautotrophy, photoheterotrophy, nitrification, and methanotrophy. The MMI was designed to complement other ongoing research at JCVI and elsewhere to characterize the microbial biodiversity of marine and terrestrial environments through metagenomic profiling of environmental samples.

## Classification and features

### 16S rRNA analysis

A representative genomic 16S rRNA sequence of strain DFL-11^T^ was compared using NCBI BLAST [4,5] using default settings (e.g., considering only the high-scoring segment pairs (HSPs) from the best 250 hits) with the most recent release of the Greengenes database [6] and the relative frequencies of taxa and keywords (reduced to their stem [7]) were determined, weighted by BLAST scores. The most frequently occurring genera were *Stappia* (36.9%), *Pannonibacter* (19.6%), *Pseudovibrio* (18.8%), *Labrenzia* (10.8%) and *Achromobacter* (5.0%) (98 hits in total). Regarding the seven hits to sequences from other members of the genus, the average identity within HSPs was 97.3%, whereas the average coverage by HSPs was 96.4%. Among all other species, the one yielding the highest score was *Stappia alba* (AJ889010) (since 2007 reclassified as *L. alba* [1]), which corresponded to an identity of 98.2% and an HSP coverage of 99.9%. (Note that the Greengenes database uses the INSDC (= EMBL/NCBI/DDBJ) annotation, which is not an authoritative source for nomenclature or classification.) The highest-scoring environmental sequence was AY701471 (Greengenes short name 'dinoflagellate symbiont clone GCDE08 W'), which showed an identity of 99.8% and an HSP coverage of 99.6%. The most frequently occurring keywords within the labels of all environmental samples which yielded hits were 'coral' (5.4%), 'microbi' (3.2%), 'marin' (3.0%), 'diseas' (2.8%) and 'healthi' (2.8%) (150 hits in total). The most frequently occurring keywords within the labels of those environmental samples which yielded hits of a higher score than the highest scoring species were 'coral' (11.1%), 'dinoflagel, symbiont' (5.7%), 'aquarium, caribbean, chang, dai, disease-induc, faveolata, kept, montastraea, plagu, white' (5.6%) and 'habitat, microbi, provid, threaten' (5.5%) (4 hits in total). These terms partially correspond with the known ecology of *L. alexandrii*.

**Figure 1:**
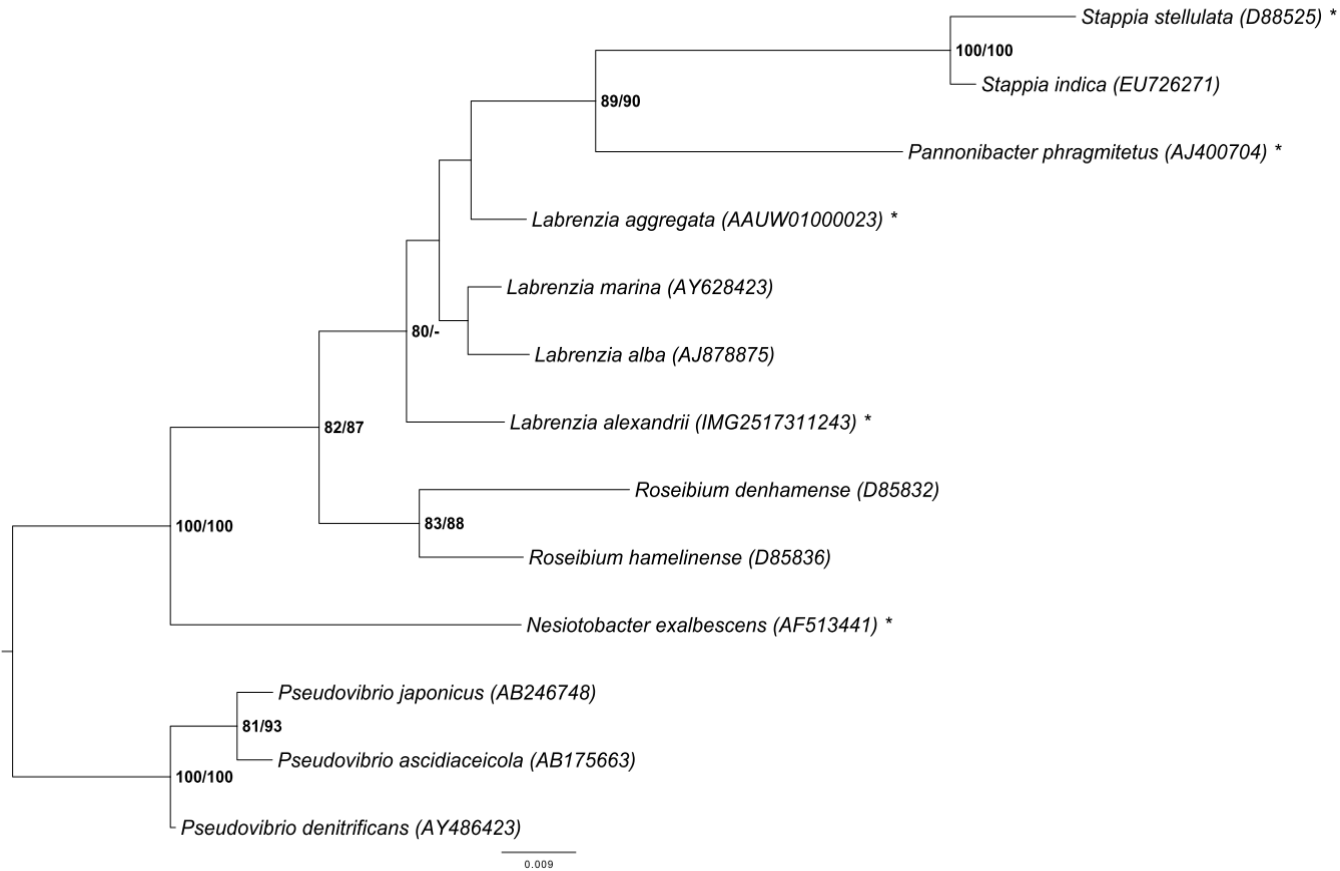
Phylogenetic tree highlighting the position of *L. alexandrii* relative to the type strains of the species of selected genera (see [1,3] and the results of the Greengenes database search described above) within the family *Rhodobacteraceae*. These genera form a clade [1,3], but it might be better not to place them in this family [3]. The tree was inferred from 1,366 aligned characters [8,9] of the 16S rRNA gene sequence under the maximum likelihood (ML) criterion [10] and rooted with *Pseudovibrio*. The branches are scaled in terms of the expected number of substitutions per site (see size bar). Numbers adjacent to the branches are support values from 1,000 ML bootstrap replicates [11] (left) and from 1,000 maximum-parsimony bootstrap replicates [12] (right) if larger than 60%. Lineages with type-strain genome sequencing projects registered in GOLD [13] are labeled with one asterisk.

Figure 1 shows the phylogenetic neighborhood of *L. alexandrii* in a 16S rRNA based tree. The sequences of the three identical 16S rRNA gene copies in the genome do not differ from the previously published 16S rRNA sequence (AJ582083).

### Morphology and physiology

**Figure 2:**
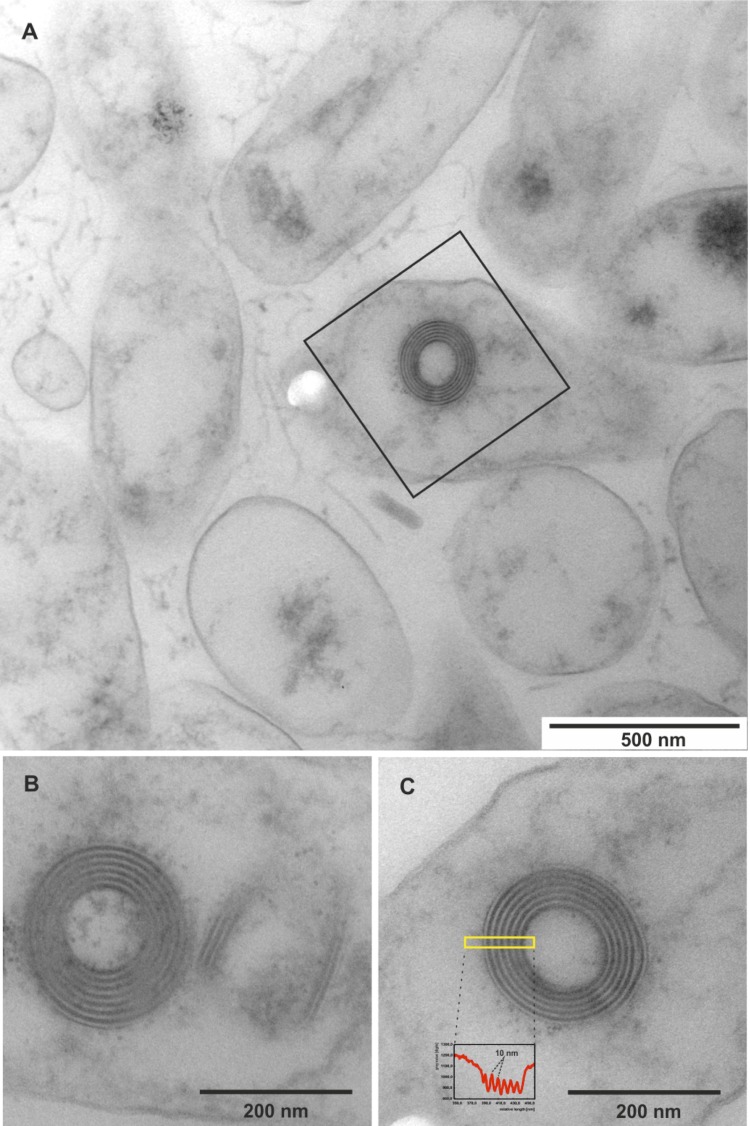
Ultrastructure of *L. alexandrii* DFL-11^T^ and its R-bodies. (A) Survey view of the cells from the near-surface position of a colony. Many bacterial remnants are visible, one of which contains an R-body; such bodies are shown enlarged in (B) and (C). (B) A pair of R-bodies, oriented at right angle towards each other, one as a cross-section and the other one cut oblique-longitudinally. The bipartite, black-white organization of the spiral layers is shown, and the averaged intensity profile (C, inset) of the boxed area shows a regular spacing of 10 nm.

The rod-shaped cells of strain DFL-11^T^ are 0.5 to 0.7 μm in width and 0.9 to 3.0 μm long with often unequal ends (Table 1 and Figure 2A), suggesting a polar mode of cell division which is increasingly being discovered in *Alphaproteobacteria* and thought to be ancient [23]. Motility is present by means of a single subpolar flagellum [1]. Star-shaped aggregated clusters occur [1]. The colonies exhibit a beige to slightly pink color [1]. Strain DFL-11^T^ has a chemotrophic lifestyle; no fermentation occurs under aerobic or anaerobic conditions [1]. Optimal growth occurs in the presence of 1-10% NaCl and pH 7.0-8.5 at 26°C, whereas no growth occurs in the absence of NaCl or of biotin and thiamine as growth factors [1]. Several organic acids like acetate, butyrate, malate and citrate as well as glucose and fructose are metabolized, but methanol, ethanol and glycerol are not used for growth [1]. Whereas gelatin is hydrolyzed by the cells, starch is not; nitrate is not reduced [1]. The strain shows a weak resistance to potassium tellurite [1].

**Table 1:** Classification and general features of *L. alexandrii* DFL-11^T^ according to the MIGS recommendations [14].

**MIGS ID**	**Property**	**Term**	**Evidence code**
	Classification	Domain *Bacteria*	TAS [15]
		Phylum *Proteobacteria*	TAS [16]
		Class *Alphaproteobacteria*	TAS [17,18]
		Order *Rhodobacterales*	TAS [17,19]
		Family *Rhodobacteraceae*	TAS [17,20]
		Genus *Labrenzia*	TAS [1]
		Species *Labrenzia alexandrii*	TAS [1]
MIGS-7	Subspecific genetic lineage	Strain DFL-11	TAS [1]
	Gram stain	Gram-negative	TAS [1]
	Cell shape	rod-shaped	TAS [1]
	Motility	motile	TAS [1]
	Sporulation	not reported	
	Temperature range	mesophile	TAS [1]
	Optimum temperature	26°C	TAS [1]
	Salinity	1–10 % (w/v) sea salt	TAS [1]
MIGS-22	Relationship to oxygen	aerobe	TAS [1]
	Carbon source	acetate, butyrate and malate	TAS [1]
	Energy metabolism	photoheterotroph	TAS [1]
MIGS-6	Habitat	marine	TAS [1]
MIGS-6.2	pH	6–9.2	TAS [1]
MIGS-15	Biotic relationship	host-associated	TAS [1]
MIGS-14	Known pathogenicity	none	TAS [1]
MIGS-16	Specific host	*Alexandrium lusitanicum*	TAS [1]
MIGS-18	Health status of host	not reported	
	Biosafety level	1	TAS [21]
MIGS-19	Trophic level	not reported	
MIGS-23.1	Isolation	ME207	TAS [1]
MIGS-4	Geographic location	not reported	
MIGS-5	Time of sample collection	April 1, 2002	TAS [1]
MIGS-4.1	Latitude	54.133	TAS [1]
MIGS-4.2	Longitude	7.867	TAS [1]
MIGS-4.3	Depth	not reported	
MIGS-4.4	Altitude	not reported	

Evidence codes – TAS: Traceable Author Statement (i.e., a direct report exists in the literature); NAS: Non-traceable Author Statement (i.e., not directly observed for the living, isolated sample, but based on a generally accepted property for the species, or anecdotal evidence). Evidence codes are from the Gene Ontology project [22].

The utilization of carbon compounds by *L. alexandrii* DSM 17067^T^ was also determined for this study using PM01 microplates in an OmniLog phenotyping device (BIOLOG Inc., Hayward, CA, USA). The microplates were inoculated at 28°C with a cell suspension at a cell density of approximately 85% Turbidity and dye D. Further additives were artificial sea salts, vitamins, trace elements and NaHC0_3_. The exported measurement data were further analyzed with the opm package for R [24], using its functionality for statistically estimating parameters from the respiration curves such as the maximum height, and automatically translating these values into negative, ambiguous, and positive reactions. The strain was studied in six independent biological replicates, and reactions with a distinct behavior between the repetitions were regarded as ambiguous and are not listed below.

*L. alexandrii* DSM 17067^T^ was positive for glycerol, D-xylose, D-mannitol, L-glutamic acid, D,L-malic acid, D-ribose, D-fructose, D-glucose, α-keto-glutaric acid, α-keto-butyric acid, uridine, L-glutamine, α-hydroxy-butyric acid, myo-inositol, fumaric acid, propionic acid, glycolic acid, inosine, tricarballylic acid, L-threonine, D-malic acid, L-malic acid and pyruvic acid. The strain was negative for D-saccharic acid, D-galactose, D-alanine, D-trehalose, dulcitol, D-serine, L-fucose, D-glucuronic acid, D-gluconic acid, D,L-α-glycerol-phosphate, sodium formate, D-glucose-6-phosphate, D-galactonic acid-γ-lactone, tween 20, L-rhamnose, D-maltose, L-asparagine, D-aspartic acid, D-glucosaminic acid, 1,2-propanediol, tween 40, α-methyl-D-galactoside, α-D-lactose, lactulose, sucrose, m-tartaric acid, α-D-glucose-1-phosphate, D-fructose-6-phosphate, tween 80, α-hydroxy-glutaric acid-γ-lactone, β-methyl-D-glucoside, adonitol, maltotriose, 2'-deoxy-adenosine, adenosine, gly-asp, D-threonine, bromo-succinic acid, mucic acid, D-cellobiose, glycyl-L-glutamic acid, L-alanyl-glycine, acetoacetic acid, N-acetyl-β-D-mannosamine, methyl pyruvate, tyramine, D-psicose, glucuronamide, L-galactonic acid-γ-lactone, D-galacturonic acid and β-phenylethylamine.

In an electron microscopic survey colonies of strain DFL-11^T^, grown on half-strength MB (Roth CP73.1) agar plates, were fixed with 2.5% glutardialdehyde, 10 mM Hepes, pH 7.1, and embedded in Spurr's epoxide resin as described in detail elsewhere [25]. Ultrathin sections (90 nm) were analyzed in the elastic bright-field mode with an energy-filter transmission electron microscope (TEM) (Libra 120 plus; Zeiss, Oberkochen), and micrographs were recorded with a 2k × 2k cooled CCD-camera (SharpEye; Tröndle, Moorenweis, Germany) at a magnification range of 4000 × to 25000 ×.

TEM analysis showed that individual cells of strain DFL-11^T^, assembled in clusters, contained refractile inclusion bodies, known as R-bodies [26,27], when plate-grown bacteria were embedded as microcolonies of different growth states. R-bodies are highly insoluble protein ribbons coiled to form a hollow cylinder within the cytoplasma of the bacterial cells [26,27]. In strain DFL-11^T^ these unusual structures were generally observed in cell remnants, which contained only small amounts of cytoplasmic material (Figure 2A). They were built mainly as five- to six-layered spirals and often had a loose electron-dense, amorphous matrix. In concentric cross- or longitudinal sections the individual layers appeared to be composed of an electron-dense dark and an electron-translucent bright layer; each doublet was found to have an average thickness of 10.1 nm (standard deviation: 0.7 nm; N = 16), ranging from minimal 8.7 nm to maximum 11.9 nm. The overall diameter of the R-bodies ranged from 183 nm to 242 nm, which is in good accordance with the dimensions of furled R-body ribbons reviewed in [27].

To date only a few bacterial species are known to produce R-bodies [26,27]. They were first described in members of the genus *'Caedibacter'*. These bacteria live as obligate endosymbionts in *Paramecium* species and confer the so-called “killer trait” to their hosts: “killer-phenotype” paramecia release *'Caedibacter'* cells *via* their cytopyge into the environment and these kill sensitive paramecia (i.e. '*Caedibacter'*-free ciliates) after being ingested. The toxic effect of *'Caedibacter'* is strictly correlated with R-body synthesis. Once incorporated into sensitive paramecia, the R-body extrudes in a telescopic fashion, thereby disrupting the bacterial cell. Cellular components are subsequently released into the cytoplasma of *Paramecium*, finally causing the ciliate’s death. It has been proposed that a lethal toxin is involved in this process, but it has not been identified so far [28]. Interestingly, a phylogenetic study based on comparative 16S rRNA gene sequencing revealed that *'Caedibacter'* is a polyphyletic assemblage, comprising *Gammaproteobacteria* related to *Francisella tularensis* as well as *Alphaproteobacteria* affiliated with *Rickettsiales* (including the obligate *Paramecium* endosymbiont '*Holospora'*) [29]. In addition to the obligate endosymbionts, some free-living bacteria, i.e. *Hydrogenophaga taeniospiralis*, *Acidovorax avenae subsp. avenae* (both *Burkholderiales*), *Rhodospirillum centenum*, an anoxygenic phototrophic alphaproteobacterium, and *Marinomonas mediterranea,* a marine gammaproteobacterium, were observed to produce R-bodies [30].

### Chemotaxonomy

Ubiquinone 10 was found as the single respiratory lipoquinone, which is a common feature in most *Alphaproteobacteria*. The spectrum of polar lipids consists of phosphatidylglycerol, diphosphatidylglycerol, phosphatidylethanolamine, phosphatidylcholin, phosphatidylmono-methylethanolamine, sulphoquinovosyldiacylglyceride, as well as an unidentified aminolipid [1]. In the fatty acids spectrum is dominated by C_18 : 1ω7_ (71%) and complemented by C_20 : 1ω7_ (9.1%), C_18 : 0_ (6.5%), 11-methyl C_18:1ω6t_ (3.7%) and some hydroxy fatty acids C_14:0 3-OH_ (3.4%) and C_16:0 3-OH_ (1.5%) as well as traces of C_18 : 1ω9_ and cyclo C_21:0_ [1]. The presence of photosynthetic pigments was tested in [1] and the absorption spectrum of the acetone/methanol extract showed that bacteriochlorophyll a was present at low concentrations. Another peak at 420 and 550 nm indicated the presence of an additional photosynthetic pigment, most probably a yet unidentified carotinoid.

## Genome sequencing and annotation

### Genome project history

The genome was sequenced within the MMI supported by the Gordon and Betty Moore Foundation. Initial Sequencing was performed by the JCVI (Rockville, MD, USA) and a high-quality draft sequence was deposited at INSDC. The number of scaffolds and contigs was reduced and the assembly improved by a subsequent round of manual gap closure at HZI/DSMZ. A summary of the project information is shown in Table 2.

**Table 2:** Genome sequencing project information

**MIGS ID**	**Property**	**Term**
MIGS-31	Finishing quality	High quality draft
MIGS-28	Libraries used	Two genomic libraries: 40kb fosmid library and 3 kB pUC18 plasmid library
MIGS-29	Sequencing platforms	ABI3730
MIGS-31.2	Sequencing coverage	9.1 × Sanger
MIGS-30	Assemblers	Consed 20.0
MIGS-31.3	Contig count	6
MIGS-32	Gene calling method	Genemark 4.6b, tRNAScan-SE-1.23, Infernal 0.81
	INSDC ID	Final ID pending; previous version ACCU00000000
	Genbank Date of Release	N/A
	GOLD ID	Gi01459
	NCBI project ID	19367
	Database: IMG	2517287006
MIGS-13	Source Material Identifier	DSM 17067
	Project relevance	Environmental, Marine Microbial Initiative

### Growth conditions and DNA extractions

A culture of DSM 17067 was grown for two to three days on a LB & sea-salt agar plate, containing (l^-1^) 10 g tryptone, 5 g yeast extract, 10 g NaCl, 17 g sea salt (Sigma-Aldrich S9883) and 15 g agar. A single colony was used to inoculate LB & sea-salt liquid medium and the culture was incubated at 28°C on a shaking platform. The genomic DNA was isolated using the Qiagen Genomic 500 DNA Kit (Qiagen 10262) as indicated by the manufacturer. DNA quality and quantity were in accordance with the instructions of the genome sequencing center.

### Genome sequencing and assembly

The genome was sequenced with the Sanger technology using a combination of two libraries. All general aspects of library construction and sequencing performed at the JCVI can be found on the JCVI website. Base calling of the sequences were performed with the phredPhrap script using default settings. The reads were assembled using the phred/phrap/consed pipeline [31]. The last gaps were closed by adding new reads produced by recombinant PCR and PCR primer walks. In total 21 reads were required for gap closure and improvement of low quality regions. The final consensus sequence was built from 60,668 Sanger reads (9.1 × coverage).

### Genome annotation

Gene prediction was carried out using GeneMark as part of the genome annotation pipeline in the Integrated Microbial Genomes Expert Review (IMG-ER) system [32]. To identify coding genes, Prodigal [33] was used, while ribosomal RNA genes within the genome were identified using the tool RNAmmer [34]. Other non-coding genes were predicted using Infernal [35]. Manual functional annotation was performed within the IMG platform [32] and the Artemis Genome Browser [36].

## Genome properties

The genome statistics are provided in Table 3 and Figures 3a, 3b and 3c. The genome consists of a 5,299,280 bp long chromosome and two plasmids with 68,647 bp and 93,929 bp length, respectively, with a G+C content of 56.4%. Of the 5,144 genes predicted, 5,071 were protein-coding genes, and 73 RNAs; pseudogenes were not identified. The majority of the protein-coding genes (81.0%) were assigned a putative function while the remaining ones were annotated as hypothetical proteins. The distribution of genes into COGs functional categories is presented in Table 4.

**Table 3:** Genome Statistics

**Attribute**	**Value**	**% of Total**
Genome size (bp)	5,461,856	100.00
DNA coding region (bp)	4,871,168	89.19
DNA G+C content (bp)	3,080,828	56.41
Number of replicons	3	
Extrachromosomal elements	2	
Total genes	5,144	100.00
RNA genes	73	1.42
rRNA operons	3	
tRNA genes	52	1.01
Protein-coding genes	5,071	98.58
Pseudo genes	0	
Genes with function prediction	4,168	81.03
Genes in paralog clusters	1,866	36.28
Genes assigned to COGs	4,140	80.48
Genes assigned Pfam domains	4,203	81.71
Genes with signal peptides	1,147	22.30
Genes with transmembrane helices	1,264	24.57
CRISPR repeats	0	

**Figure 3a:**
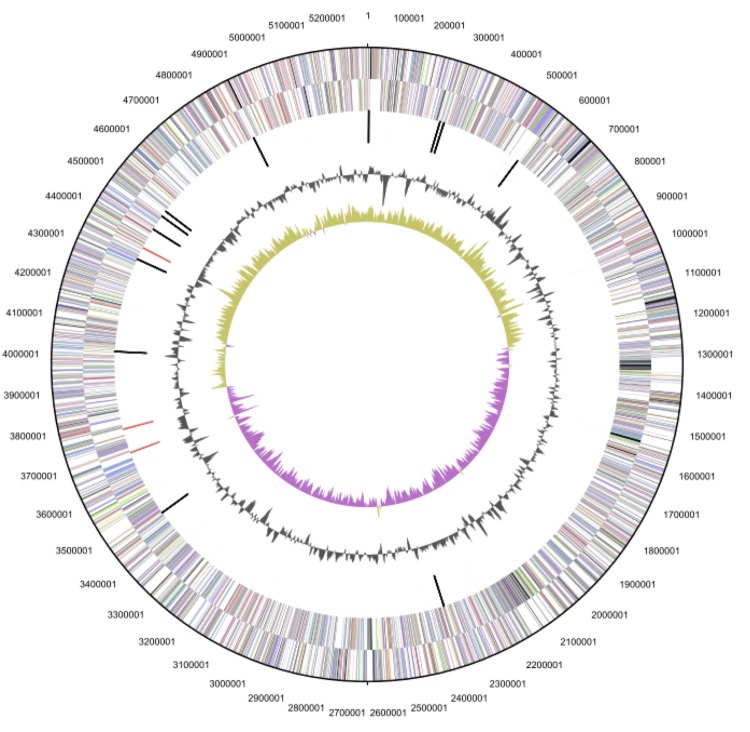
Graphical map of the chromosome. From outside to the center: Genes on forward strand (color by COG categories), Genes on reverse strand (color by COG categories), RNA genes (tRNAs green, rRNAs red, other RNAs black), GC content, GC skew.

**Figure 3b:**
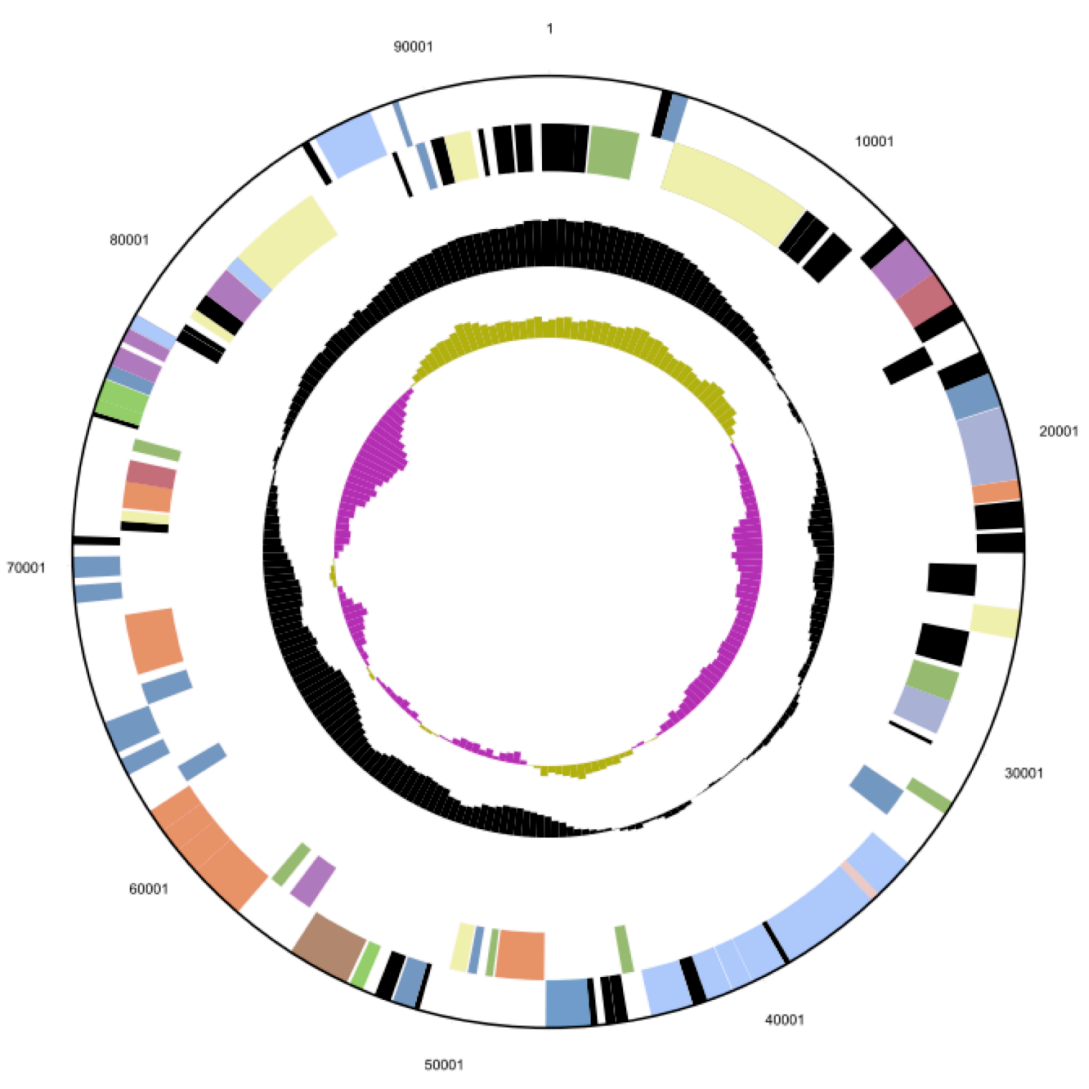
The larger of the two plasmids (LADFL_5, not drawn to scale with the chromosome). From outside to the center: Genes on forward strand (color by COG categories), genes on reverse strand (color by COG categories), RNA genes (tRNAs green, rRNAs red, other RNAs black), GC content, GC skew.

**Figure 3c:**
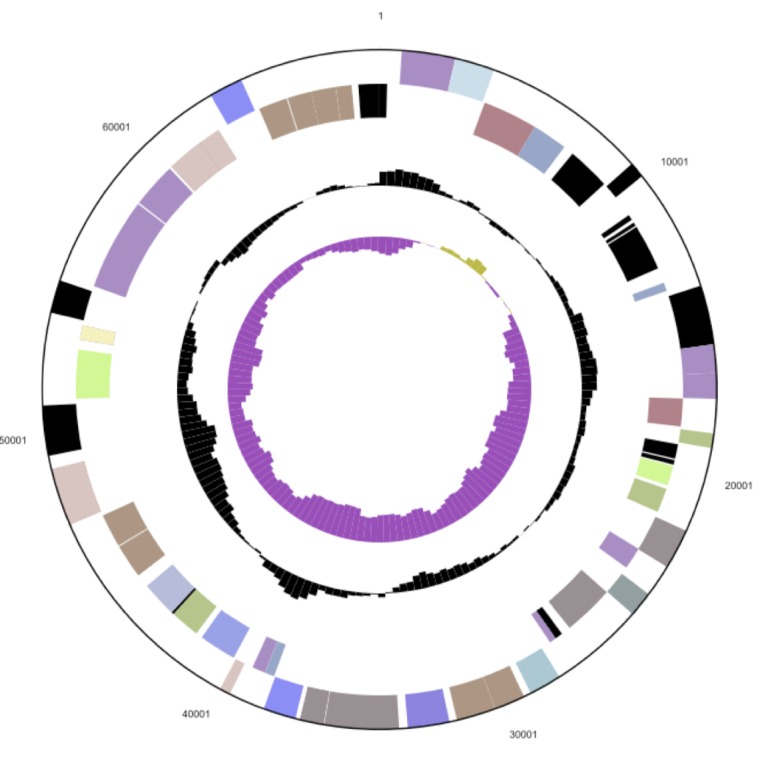
The smaller of the two plasmids (LADFL_6, not drawn to scale with the chromosome). From outside to the center: Genes on forward strand (color by COG categories), Genes on reverse strand (color by COG categories), RNA genes (tRNAs green, rRNAs red, other RNAs black), GC content, GC skew.

**Table 4:** Number of genes associated with the general COG functional categories

**Code**	**Value**	**%age**	**Description**
J	179	3.88	Translation, ribosomal structure and biogenesis
A	2	0.04	RNA processing and modification
K	363	7.86	Transcription
L	133	2.88	Replication, recombination and repair
B	3	0.06	Chromatin structure and dynamics
D	38	0.82	Cell cycle control, cell division, chromosome partitioning
Y	0	0	Nuclear structure
V	51	1.10	Defense mechanisms
T	330	7.15	Signal transduction mechanisms
M	223	4.83	Cell wall/membrane/envelope biogenesis
N	115	2.49	Cell motility
Z	2	0.04	Cytoskeleton
W	0	0	Extracellular structures
U	91	1.97	Intracellular trafficking, secretion, and vesicular transport
O	167	3.62	Posttranslational modification, protein turnover, chaperones
C	260	5.63	Energy production and conversion
G	276	5.98	Carbohydrate transport and metabolism
E	518	11.22	Amino acid transport and metabolism
F	91	1.97	Nucleotide transport and metabolism
H	174	3.77	Coenzyme transport and metabolism
I	186	4.03	Lipid transport and metabolism
P	232	5.02	Inorganic ion transport and metabolism
Q	136	2.95	Secondary metabolites biosynthesis, transport and catabolism
R	582	12.61	General function prediction only
S	465	10.07	Function unknown
-	1,004	19.52	Not in COGs

## Insights into the genome

### R-body genes

In '*Caedibacter taeniospiralis'*, three genes (*rebA*, *rebB* and *rebC*) were identified to determine the R-body production. They are clustered on large plasmids, ranging from 41-49 kb, and encompass 345 bp, 318 bp and 171 bp (accession number U04524), respectively. The corresponding proteins RebA (114 aa, 18 kDa), RebB (105 aa, 13 kDa) and RebC (56aa, 10 kDa) are necessary to assemble R-bodies through polymerization processes [37]. Furthermore, a putative forth gene *rebD* (249 bp; RepD 82aa) is located between *rebB* and *rebC* and might be involved in R-body formation.

Based on high sequence similarities to the *C. taeniospiralis* R-body protein RebB, three homologues (ladfl_0000085, ladfl_0000090 and ladfl_0000091) were detected on the chromosome of strain DFL-11^T^. Their amino acid sequence length is 122 aa, 109 aa and 76 aa, respectively, which is in accordance with R-body proteins found in *C. taeniospiralis* 47, and they were all assigned to the Pfam family RebB (PF11747). The chromosomal arrangement of R-body genes in strain DFL-11^T^ is not contiguous; ladfl_0000085 is separated from ladfl_0000090 and ladfl_0000091 by four hypothetical genes (ladfl_0000086 -ladfl_0000089). Interestingly, a putative alternative sigma-factor of the ECF subfamily (ladfl_0000084, upstream of ladfl_0000085) flanks the R-body gene cluster, indicating that *reb* gene expression in strain DFL-11^T^ is regulated by extracytoplasmic stimuli. Gene arrangements orthologous to the *L. alexandrii* DFL-11^T^ *reb* gene cluster were found in the alphaproteobacteria *Roseibium sp.* TrichSKD4 (NZ_GL47637) and *Polymorphum gilvum* (NC_015259), organisms which are closely related to *L. alexandrii* [38].

### Plasmids

Genome sequencing of *L. alexandrii* DSM 17067^T^ reveals the presence of two RepABC-type plasmids designated LADFL_5 and LADFL_6 with sizes of 93,929 bp and 68,647 bp, respectively. This outcome is in agreement with a previous study about the genome organization of different marine *Alphaproteobacteria* including DFL-11^T^ [39]. Pulsed-field gel electrophoresis (PFGE) showed faint bands with estimated sizes of 88 kb and 65 kb, and their circular conformation has been documented by comparative analyses with distinct PFGE parameters. An additional linear fragment of about 35 kb, which has not been recovered by genome sequencing, may represent a prophage (see below) whose excision from the genome depends on the cultivation conditions. Both plasmids represent RepABC-type replicons with the partitioning genes *repA* and *repB* as well as the replicase *repC* that are located in a typical operon [40]. Phylogenetic analyses of the replicases provides the basis for the classification of alphaproteobacterial plasmids [41]. The respective phylogeny of both RepC sequences from *L. alexandrii* DSM 17067^T^ (ladfl_05027, ladfl_05140) documents a close affiliation with rhizobial genes to an exclusion of sequences from *Rhodobacterales* that are located in distinct subtrees (data not shown [42] ). Both plasmids seem to be equipped with characteristic post segregational killing systems consisting of a toxin/antitoxin operon that prevent plasmid loss (ladfl_05100/ladfl_05101, ladfl_05128/ladfl_05129 [43] ).

Plasmid LADFL_5 contains several genes that are related to heavy-metal resistance [44] and eight of them are related to the COG category “Inorganic ion transport and metabolism” (see also Table 4). This set includes the *mer*-operon composed of *merR*, *merT*, *merF* and mercuric reductase MerA, which are part of the Gram-negatives' mercury-resistance system [45]. This plasmid also harbors a predicted P-type ATPase translocating heavy-metal ions and components of a Cd2+, Zn2+ or Co2+ efflux system. The resistance to a wide pallet of heavy-metal ions may enable the strain to dwell in polluted environments [44]. The second conspicuous trait of LADFL_5 is the presence of a complete type-IV secretion system (T4SS [46] ). The *virB* operon (ladfl_05033 to ladfl_05043) is required for the formation of a functional transmembrane channel and pilus formation. Moreover, the *virD* gene cluster including the characteristic DNA relaxase (ladfl_05091) and the coupling protein VirD4 (ladfl_05093) indicates that the T4SS machinery represents a functional conjugation system. The lysozyme TraH_2 (ladfl_05088), which is required for the degradation of the peptidoglycan cell wall and transmembrane channel formation, is annotated as specific protein of *Rhizobiales*, an affiliation that is in agreement with the outcome of the phylogenetic RepC analysis [42].

Plasmid LADFL_6 is dominated by more than a dozen genes that are involved in sugar metabolism. It contains the complete operon for the conversion of glucose-1-phosphate into dTDP-L-rhamnose (*rmlC*, *rmlD*, *rmlA*, *rmlB*) that is a common component of the cell wall and capsule of many pathogenic bacteria [47]. Three glycosyltransferases, some components of an ABC-type polysaccharide transport system as well as a sugar transferase for lipopolysaccharide synthesis and a lipid A core O-antigen ligase (ladfl_05144, ladfl_05145) are indicative for a functional role of the plasmid for exopolysaccharide formation. Extracellular polysaccharids of the Sym plasmid are required for root hair attachment in *Rhizobium leguminosarum* [48] and the plasmid LADFL_6 may also be required for biofilm generation. This prediction is compatible with the origin of strain DFL-11^T^ that has been isolated from the dinoflagellate *A. lusitanicum* [1].
